# La rupture intra péritonéale d’un kyste hydatique au cours de la grossesse: à propos d’un cas rare

**Published:** 2012-01-09

**Authors:** Myriam Rachad, Fatim Zahra Fdili, Ouafae Slimani, Hikmat Chaara, Hakima Bouguern, Moulay Adbelillah Melhouf

**Affiliations:** 1 Service de gynécologie-obstétrique II, CHU Hassan II, Fès, Maroc

**Keywords:** Kyste hydatique, foie, pelvis, grossesse, chirurgie, Maroc

## Abstract

Le kyste hydatique est une parasitose qui sévit à l’état endémique au Maroc. Cependant sa rupture au cours de la grossesse reste rare. Nous rapportons le cas d’une patiente de 23 ans, admise aux urgences en état de choc avec bouffissure du visage sur aménorrhée de 8 semaines, chez laquelle le diagnostic d’hydatidose à la fois hépatique et pelvienne, avec éventuelle rupture de l’un des kystes hépatiques a été posé par la radiologie. Après mesures de réanimation, le traitement chirurgical a été instauré, avec bonne évolution. Nous essayons à partir de ce cas et à travers une revue de littérature, de préciser les difficultés diagnostiques et thérapeutiques rencontrées dans la prise en charge des kystes hydatiques, surtout compliquées, au cours de la grossesse.

## Introduction

L’échinococcose hydatique ou kyste hydatique est une helminthiase provoquée par le développement chez l’Homme de la forme larvaire d’un tænia du chien *Echinococcus granulosus*.

Sa gravité tient essentiellement à ses complications dominées par la rupture dans les voies biliaires, l’infection, et la rupture intra péritonéale. Cette dernière reste une complication rare, qui réalise une véritable urgence, et représente un tournant évolutif péjoratif de l’hydatidose : dans l’immédiat, par le risque du choc anaphylactique et de la péritonite hydatique, secondairement, par l’hydatidose péritonéale secondaire.

La découverte du kyste hydatique du foie (KHF) et surtout d’hydatidose multiple au cours de la grossesse est une situation assez rare, qui pose à la fois un problème diagnostique, thérapeutique et pronostic.

## Observation

Il s’agit de Mme M.J de 23 ans, 3^ème^ geste, 2^ème^ pare, sans antécédents particuliers, notamment pas de notion de traumatisme, à 8 semaines d’aménorrhées(SA), admise aux urgences gynéco- obstétricales pour lipothymie, vomissements avec bouffissure du visage.

L’examen général a objectivé : une tension artérielle à 7/5, un pouls à 100 b/min, une fréquence respiratoire à 30 c/min, avec bouffissure du visage manifeste et des extrémités froides.

**Figure 1:**
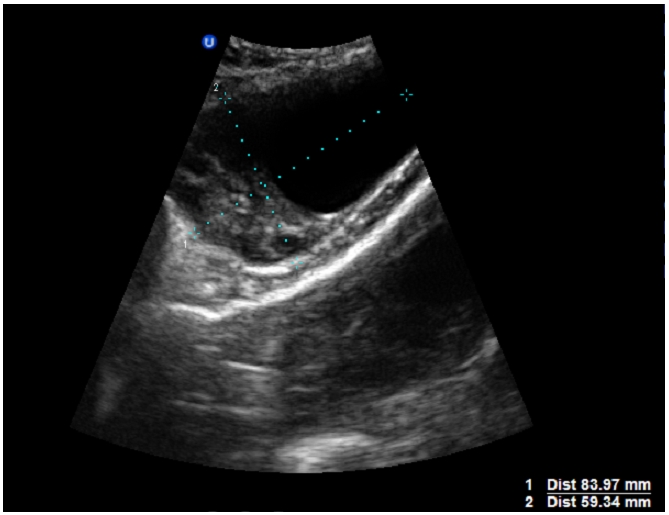
Image d’échographique abdomino-pelvienne, montrant chez notre patiente de 23 ans, admise pour état de choc sur aménorrhée de 8 semaines, la présence au niveau de la fosse iliaque gauche d’une image hétérogène de 83 mm de grand axe, bien limitée, rappelant une lésion annexielle

**Figure 2:**
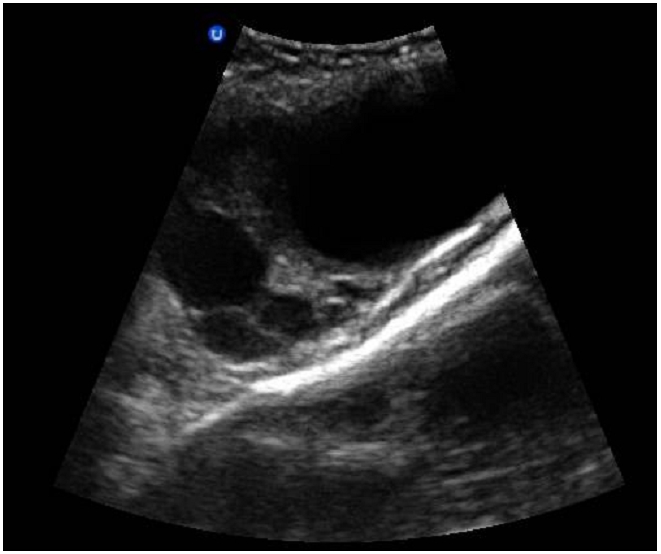
Image d’échographique abdomino-pelvienne, montrant chez notre patiente de 23 ans, admise pour état de choc sur aménorrhée de 8 semaines, l’aspect multi cloisonné de la lésion de la fosse iliaque gauche

**Figure 3:**
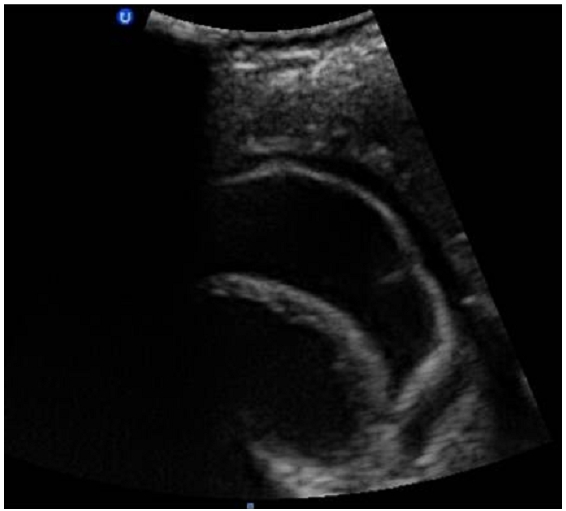
Image d’échographique abdominale, montrant chez notre patiente de 23 ans, admise pour état de choc sur aménorrhée de 8 semaines, un kyste hépatique du segment III affaissé, avec membrane décollée témoignant de sa rupture et faisant évoquer en premier selon le contexte et les autres lésions hépatiques associées, un kyste hydatique rompu

La palpation abdomino-pelvienne a révélé la présence d’une sensibilité abdominale diffuse, avec masse ferme de la fosse iliaque gauche faisant 10cm, fixe et sensible. L’examen gynécologique a mis en évidence un utérus légèrement augmenté de taille sensible à la mobilisation. Après mise en condition de la patiente : deux voies veineuses périphériques, remplissage au sérum salé. Une échographie pelvienne réalisée en premier a révélé une grossesse intra utérine évolutive de 8 SA, avec présence en latéro utérin gauche d’une image hétérogène multi cloisonnée faisant 9cm de grand axe, faisant évoquée une masse annexielle (Figure 1 et Figure 2). Le complément de l’exploration abdominale a objectivé 3 images kystiques hépatiques multicloisonées au niveau des segments III, VI, IIX, évocatrices d’une hydatidose du foie, associées à un kyste hydatique pelvien.Le kyste du segment III était affaissé, avec membrane décollée évoquant une rupture (Figure 3).

Après stabilisation de l’état hémodynamique, la patiente a bénéficié d’une laparotomie qui a mis en évidence les 3 kystes hépatiques, dont celui du segment III rompu, avec kyste pelvien au dépend du péritoine pariétal et un épanchement intrapéritonéal de faible abondance.

Le geste opératoire a consisté, après lavage et protection de la cavité péritonéale, à une résection du dôme saillant du kyste du segment III et VI. La ponction du kyste du segment IIX a ramené du sang franc, d’où la décision de le respecter vu la possibilité de communication veineuse notamment sus hépatique. Le kyste pelvien a bénéficié également d’une résection du dôme saillant avec mise en place d’une sonde de Sallem. L’évolution post opératoire était sans particularité, notamment la grossesse toujours évolutive. Le complément par un traitement médical, fortement indiqué dans cette situation, n’a pas été instauré, vu la contre-indication absolue de l’albendazole chez la femme enceinte surtout au premier trimestre. La patiente est soumise à une surveillance clinique et échographique. L’albendazole est prévu en post-partum.

## Discussion

L’hydatidose est une anthropozoonose cosmopolite. Au Maroc, cette affection sévit à l’état endémique. Elle touche l’Homme quel que soit l’âge et le sexe. Elle atteint tous les viscères sans exception avec un tropisme pour le foie. La rupture aiguë de kyste hydatique dans le péritoine est une complication rare de l’hydatidose. Sa fréquence varie de 1 à 2 %, selon les séries de la littérature [1]. Peu de cas de rupture sont décrits au cours de la grossesse.

En effet, un traumatisme abdominal fermé est à l’origine de 12 à 35 % des KH rompus. Ce traumatisme peut être un accident de la voie publique, domestique, sportif ou autre. Le point d’impact peut être en regard du KH ou à distance et c’est l’onde de choc qui sera transmise au KH sous tension. L’effort physique peut aussi déclencher la rupture [1]. La rupture est dite spontanée lorsqu’il n’y a pas de facteur déclenchant, comme le cas de notre patiente.

Cliniquement la rupture aiguë du KH dans le péritoine se caractérise par son début brutal et son polymorphisme allant du tableau de la péritonite aigue, dont la fréquence est estimée à 82% dans la série de Beyrouti [1], 91% dans la série de Zaouche [2], à un choc anaphylactique, éventualité certaine mais qui reste rare. Un seul cas décrit dans la série de Beyrouti [1].

En imagerie, l’échographie abdominale représente le meilleur examen complémentaire permettant de préciser : le siège du kyste, son diamètre, sa stadification, mais aussi de guetter la rupture, par la présence d’un kyste hydatique affaissé avec une membrane décollée, associés à un épanchement péritonéal [2–4].

Les avantages de la tomodensitométrie par rapport à l’échographie sont [4–6] : Une meilleure identification des aspects échographiques peu spécifiques tels que les types I et IV de la classification de Gharbi; l’étude aisée des kystes hydatiques calcifiés en totalité ou partiellement ; la détermination de la taille exacte du kyste et ses rapports avec les organes voisins ; la détection des complications, en particulier la surinfection par la mise en évidence de gaz intra-kystique en post-opératoire ; l’étude de complications post-opératoires, surtout chez les patients obèses et multi-opérés ; le diagnostic de récidive.

L’imagerie par résonance magnétique (IRM) permet facilement de poser le diagnostic de rupture, mais aussi de surveiller l’évolution du kyste hydatique sous traitement médical [7–11].

La sérologie hydatique, l’une des principales investigations complémentaires, dans le diagnostic des KH, perd de son intérêt vu son retard diagnostic par rapport à l’urgence de la décision thérapeutique. Cependant, ses résultats viennent confirmer rétrospectivement le diagnostic d’hydatidose et servir de moyen de surveillance [1]. Chez la femme enceinte, le diagnostic d’une rupture péritonéale de kyste hydatique est un diagnostic inattendu, de découverte fortuite, surtout en absence d’antécédent d’hydatidose.

Le KHF chez la femme enceinte pose essentiellement un problème d’attitude thérapeutique en rapport avec le moment et le choix du procédé thérapeutique.

En absence de complications, impliquant une prise en charge urgente, le deuxième trimestre est considéré comme le meilleur moment pour opérer une femme enceinte à cause du risque moins important de fausse couche (0 contre 12 % au premier trimestre) et d’accouchement prématuré (5 à8 % contre 30 % au troisième trimestre) [12].

La chirurgie par voie classique reste pour le moment le meilleur moyen thérapeutique. La coeliochirurgie n’est plus une contre-indication au cours de la grossesse, mais cette voie d’abord n’a jamais été utilisée pour traiter le KH chez la femme enceinte [13].

Le traitement de la péritonite hydatique peut être réalisé par du sérum hypertonique. En fait, le choix du scolicide reste un sujet de controverse. Le sérum hypertonique à 20% est efficace mais peut entraîner des troubles hydro-électrolytiques parfois graves [8]. L’eau oxygénée à 10 volumes est à la fois un désinfectant et un scolicide efficace, mais elle risque d’entraîner des collapsus peropératoires. La toilette au sérum physiologique est en fait le temps essentiel du traitement de la péritonite hydatique. Elle doit être abondante avec usage d’un système aspiratif de gros calibre, permettant d’aspirer tous les éléments fertiles du KH. La technique opératoire, représentée essentiellement par la résection du dôme saillant (RDS), fait courir le risque de fistule biliaire et de suppuration des cavités résiduelles parfois suspendues ou mal drainées. Qui peuvent être responsables d’une longue hospitalisation qui elle-même potentialise le risque de surinfection nosocomiale et de septicémie. C’est ce qui souligne l’intérêt de l’effacement de la cavité kystique, après résection du dôme du kyste, par capitonnage ou par épiplooplastie, et l’intérêt du drainage [9]. Les suites opératoires immédiates sont dominées par la suppuration de la cavité résiduelle et la fistule biliaire [2,10]. Le traitement médical, albendazole est contre indiqué au cours du premier trimestre, à cause, des effets embryotoxiques et tératogènes démontrés chez l’animal [14]. Au cours du deuxième et troisième trimestre, l’innocuité de l’albendazole est encore non démontrée [14,15]. Deux complications tardives menacent à long terme l’évolution de la rupture aiguë du KH dans le péritoine et justifient une surveillance prolongée [14,16]: la récidive au niveau de l’organe primitif où siégeait le KH rompu dite récidive “in situ” ; l’hydatidose péritonéale secondaire due à une greffe sous-séreuse d’éléments fertiles du KH rompu et qui ont pu résister aux moyens de défense de l’hôte.

Le suivi lointain se fonde sur des contrôles cliniques, sérologiques et échographiques tous les 3 mois la première année, puis tous les 6 mois pendant les 2 années suivantes. Enfin tous les ans jusqu’à la 5ème année [11]. Si, à cette date, le bilan persiste négatif, la surveillance peut être arrêtée. En cas de discordance clinique, échographique et sérologique, l’examen tomodensitométrique doit être demandé. Cette stratégie de surveillance se heurte à la non-coopération des patients qui sont souvent de niveaux socio-économiques modestes. Pour cela, nous insistons sur l’importance de l’information et de la sensibilisation des patients et sur l’intérêt du suivi à long terme, vu le risque potentiel de récidive hydatique.

## Conclusion

Bien que l’hydatidose soit une affection endémique au Maroc, la rupture d’un kyste hydatique, au cours de la grossesse, reste une situation exceptionnelle. Elle pose à la fois un problème diagnostique et thérapeutique. D’où tout l’intérêt de la prévention dont le substratum essentiel est la lutte contre l’infestation de l’hôte définitif, la protection de l’hôte intermédiaire et la lutte contre la contamination de l’Homme.
